# Effect of 5β-dihydrotestosterone on vasodilator function and on cell proliferation

**DOI:** 10.1371/journal.pone.0312080

**Published:** 2024-10-23

**Authors:** David Sánchez-Fernández, Aritz Eguibar, Cristina López, Ángel M. Cuesta, Virginia Albiñana, Soline Rogers-Ezewuike, Juan A. Gómez-Rivas, Laura Saldaña, Luisa M. Botella, Mercedes Ferrer

**Affiliations:** 1 Departamento de Fisiología, Facultad de Medicina, Universidad Autónoma de Madrid, Madrid, Spain; 2 Servicio de Urología, Hospital Quirón Salud, Marbella, Spain; 3 Departamento de Biomedicina Molecular, Centro de Investigaciones Biológicas Margarita Salas, Consejo Superior de Investigaciones Científicas, Madrid, Spain; 4 CIBERER, Centro de Investigación Biomédica en Red de Enfermedades Raras, Unidad 707, Instituto de Salud Carlos III (ISCIII), Madrid, Spain; 5 Servicio de Urología, Hospital Clínico San Carlos, Madrid, Spain; 6 Departamento de Cirugía, Facultad de Medicina, Universidad Complutense de Madrid, Madrid, Spain; 7 Grupo de Fisiopatología Ósea y Biomateriales, Hospital Universitario La Paz-IdiPAZ, Madrid, Spain; 8 Centro de Investigación Biomédica en Red de Bioingeniería, Biomateriales y Nanomedicina, CIBER- BBN, Madrid, Spain; Tohoku University School of Medicine: Tohoku Daigaku Daigakuin Igakukei Kenkyuka Igakubu, JAPAN

## Abstract

Aging is one of the main factors associated with cardiovascular diseases. Androgens exert beneficial effects on the cardiovascular system and testosterone (TES) replacement therapy improves cardiometabolic risk factors. However, TES is contraindicated in patients with prostate cancer due to its proliferative effects on prostatic tumor cells. Additionally, TES and its reduced metabolites 5α- and 5β-dihydrotestosterone (5α-DHT and 5β-DHT) exert vasodilatory effects. Since androgen levels decrease during aging and 5β-DHT lacks genomic effects, this study is focused on analyzing its effect on vasodilator function and the proliferation rate of prostatic tumor and vascular smooth muscle cells. To study the vascular function, mesenteric arteries from aged-orchidectomized Sprague-Dawley rats were used. Mesenteric segments were divided into one control (without treatment) and three groups with the androgens (10 nM, 30 min) to analyze: acetylcholine- and sodium nitroprusside-induced responses and nitric oxide and superoxide anion production. To analyze cell proliferation, the effect of androgens on cell viability was determined. The results showed that 5β-DHT improves vasodilator function in arteries from aged-orchidectomized rats and induces antioxidant action, while the proliferation rate of the androgen-dependent prostatic tumor cells remains unaltered. These results make 5β-DHT a promising therapeutic agent for the treatment of cardiovascular pathologies.

## Introduction

It is well described that androgens exert protective effect on vascular function and structure by regulating the release and function of vasoactive factors, including nitric oxide (NO), prostanoids and reactive oxygen species (ROS) [1–5]. In addition, epidemiological studies in men have shown an association between lower plasma levels of testosterone (TES) and a higher incidence of cardiovascular diseases [6–8]. Furthermore, patients with advanced prostate cancer under androgen deprivation therapy (ADT), have shown increased risk factors for cardiovascular diseases [9]. These risk factors include alterations in lipid profile, insulin resistance as well as increase in levels of pro-inflammatory and pro-oxidant cell mediators [10–12]. These modifications have also been observed during the aging process, in which a decrease in testosterone levels also occurs [13]. Likewise, clinical studies have reported that TES treatment in men with a history of cardiovascular disease has shown therapeutical benefits in different cardiovascular risk parameters [13, 14]. However, TES therapy would be contraindicated in patients with prostate cancer due to the proliferative effect that TES exerts in prostate cancer cells [15]. In the last decade, special attention has been paid to the 5-dihydroreduced metabolites of TES. Different studies have described that TES and its 5-reduced dihydrometabolites, 5α- and 5β-dihydrotestosterone (5α- and 5β-DHT), induce an acute non-genomically vasorelaxation of isolated blood vessels [16–20] and also increase the vasorelaxation through different vasoactive mediators [21] which could be participating in the systemic hypotensive effect observed in vivo [22, 23]. Of particular interest is 5β-DHT since it is a metabolite genomically inactive, without androgenic properties, unlike 5α-DHT whose androgenic action is associated with prostate cancer cell proliferation [24, 25]. Additionally, 5β-DHT is a non-aromatizable androgen, therefore lacking estrogenic properties [17].

It is also well known that cardiovascular diseases are the leading cause of death worldwide [26] especially in population over 60 years of age [27]. Since life expectancy is increasing, the prevention and treatment of cardiovascular diseases are of great im-portance to improve the life quality of the elderly population. Undoubtedly, lifestyle plays an important role in the aging process, but additional therapies may also be relevant and specifically useful for patients under ADT. Based on the aforementioned properties of the 5-dihydroreduced metabolites of TES, the first objective was to investigate the effect of 5β-DHT on the vasodilator function of the mesenteric artery since this vascular bed importantly contributes to the regulation of the systemic vascular resistance [28–30]. Therefore, in mesenteric arterial segments from aged-orchidectomized rats, the endothelium-dependent and -independent relaxation as well as the production of NO and super-oxide anion were analyzed. Due to the action of androgens on vascular remodeling and on the growing of prostate cancer cells, the second objective was to analyze the possible effect on the proliferation of vascular smooth muscle cells (VSMC) as well as on androgen-dependent (LNCaP) and -independent (DU145) prostatic tumor cells.

## Materials and methods

### Animals and vascular tissue preparation

Male Sprague-Dawley (SD) rats were provided by the Animal Facility of the Universidad Autónoma de Madrid (UAM) (Registration number EX-02IU). All animal protocols were approved by the UAM Research Ethics Committee, according to directives 609/86 CEE and RD. 233/88 of the Ministerio de Agricultura, Pesca y Alimentación de España (PROEX 182.7/21). Those directives require 12 hours of light/dark cycle, 21º C of constant temperature and standard feeding with fodder and water *ad libitum*. The experiments followed the instructions described in “Guiding Principles in the Care and Use of Animals” approved by the directive 63/2010 of the European Union (UE) and Spanish regulation RD53/2013.

Male sex-hormone deprived rats were used. At 5 months of age, and under anesthesia by isofluorane inhalation, rats were subjected to bilateral orchidectomy. The animals were treated with 0.30 mg/kg meloxicam SC (Metacam 5 mg/ml; Boehringer-Ingelhelm) immediately after surgery and with 50 mg/kg ibuprofen, via oral administration for 4 days. When the rats reached 24 months of age, they were euthanized by CO_2_ inhalation and decapitation. Days before castration and euthanization, rats were weighted and their blood pressure was in-directly measured [2, 3, 21] in awake animals by the tail-cuff method using a Letica Digital Pressure Meter LE5000 (Barcelona, Spain).

Once the animals were euthanized, the upper mesenteric artery was dissected out, cleaned of adhering adipose and conjunctive tissues and placed in a Krebs-Henseleit solution (KHS) at 4º C containing (in mM): 115 NaCl, 4.6 KCl, 2.5 CaCl_2_, 1.2 KH_2_PO_4_, 1.2 MgSO_4_·7H_2_O, 25 NaHCO_3_, 0.03 NaEDTA and 11.1 glucose. The mesenteric artery was cut in segments of 4 mm of length which were divided into a control group (without andro-gens) and three other groups, incubating with each of the androgens (TES, 5α-DHT and 5β-DHT) at 10 nM for 30 min.

### Serum levels of testosterone

Serum was obtained at the time of sacrifice by collecting trunk blood, followed by centrifugation and frozen at -80ºC and used directly to perform the assay. Testosterone levels were determined using the monoclonal enzyme immunoassay kit (Cayman Chemical) as previously reported [3]. The assay was performed according to the manufacturer’s instructions. Levels of testosterone were expressed as pg/mL.

### Vascular reactivity

The method used for isometric tension recording was described by Nielsen and Owman [31] and has been widely used [1–3, 21, 31]. Briefly, the arterial segments were suspended in an organ bath containing 5 mL of KHS at 37º C and continuously bubbled with 95% of O_2_-5% CO_2_ mixture (pH 7,4). Two parallel stainless-steel pins were introduced through the arterial lumen, one fixed to the organ bath wall and the other connected to a force transducer (Grass FTO3C; Quincy, Mass, USA) which is connected to a polygraph (model 7D Grass). The mesenteric segments were subjected to 0.5 g tension which was adjusted every 15 minutes for an equilibration period of 90 min. During this time, the KHS was changed every 30 minutes. Once vessels were stabilized, they were exposed to a high potassium solution (KCl 75 mM) to check their functional integrity. Following a washout period, vascular endothelial viability was determined according to the ability of 10 μM acetylcholine (ACh) to relax precontracted segments with 0.1 μM noradrenaline (NA). Only vascular rings in which ACh-induced relaxation was greater than 60% were used. The vascular rings were immediately washed three times with KHS to recover the baseline tension.

To test the effect of the androgens on the vasodilator response, mesenteric segments were separately incubated with each androgen (10nM) for 30 min before inducing contraction with 0.1 μM of NA. Then, the vasodilator concentration-response curves to ACh (10 pM-10 μM) to observe the endothelial mediated vascular response, and to SNP (10 pM-10 μM) to observe the NO-induced vasodilator response were performed.

The relaxation induced by ACh and SNP was expressed as percentage of initial contraction elicited by NA.

### Release of nitrites

Firstly, the mesenteric arterial segments were immersed in 200 μL of KHS bubbled with carbogen for 30 minutes changing the KHS every 10 minutes and collecting the last medium to measure the basal nitrite release (b1). Following this, the arterial segments were immersed again in 200 μL but were incubated with their respective androgens (unlike control segments) at 10 nM for 30 minutes, changing the incubation medium every 10 minutes and collecting the last medium to measure the effect of the androgens in the basal nitrite release (b2). Those mediums were stored at -80 °C until its analysis. The nitrite release measurement was performed with the Nitric Oxide Assay Kit (Abcam). Results of nitrite release were expressed as the ratio of the values obtained after the incubation with each androgen divided by the values obtained before the incubation (b2/b1).

### Detection of superoxide anion

To analyze the production of superoxide anion in the absence (control) and in the presence of each androgen the fluorescent dye hydroethidine (HE) was used. The mesenteric artery segments used in the nitrite release experiments were cryoprotected into 30% (w/v) sucrose in PBS and frozen and stored at -80°C. The arteries from three animals were sliced into segments of 10 μm and mounted onto glass slides. Two or three sets of artery slices (with the four experimental conditions), belonging to the same rat, were mounted on different glass slides and processed in different days. Artery slices were washed with PBS (3 times, 10 min each) and permeabilized with PBS-T (0.01% v/v of Tween 20 diluted in PBS) for 10 minutes. Then, the mesenteric artery segments were incubated with 5 μM HE for 35 min in a light protected and humidified chamber at 37 ºC. After this, vessels were washed again with PBS (3 times, 10 min each) and mounted and covered with Citifluor. Finally, the slides were imaged using an 40x oil immersion objective on a laser scanning confocal microscope LEICA (TCS ST2 DM IRE2) with the excitation wavelength set at 488 nm and emission wavelength at 610 nm. Laser and image settings were unchanged for the acquisition of images from the four groups of arteries. The photomicrographs show the intensity and location of HE, which reflects superoxide production, so that comparison of control and androgen incubated groups could be made. To analyze fluorescence intensity the ImageJ Analysis Software (National Institutes of Health [NIH], RRID: SCR_003070) was used. Three or four sections of each arterial slice area in each of the four experimental conditions (Control, TEST, 5α- and 5β-DHT) were measured on the maximal projection and the respective averages were made. The amount of superoxide anion formation was expressed as arbitrary units (A.U.) of fluorescence emitted by HE normalized with the area and relative to the control condition (= 100).

### Cell cultures

Vascular smooth muscle cells (SV40LT-VSMC) and androgen-dependent prostate tumor cells (LNCaP) were obtained from the American Type Culture Collection (ATCC). Androgen-independent prostate cancer cell line (DU145) was kindly provided by Dr. Botella’s lab (Centro de Investigaciones Biológicas Margarita Salas, CSIC). Cells were grown in Dulbecco’s Modified Eagle’s Medium (DMEM) supplemented with 10% (v/v) fetal bovine serum (FBS), 2 mM L-glutamine and 100 U/mL penicillin/streptomycin (GIBCO, Grand Island, NY, USA) at 37ºC in a humidified atmosphere containing 5% CO_2_. Cells were seeded and allowed for the attachment followed by the androgen treatment (at different concentration and incubation time). Some experiments were performed using a hormone-free serum, lipid-depleted FBS (HyClone), to avoid interference of the FBS hormone content with the results.

The effect of androgens on cell proliferation was expressed as percentage of cell viability with respect to the control condition. The viability assay was performed by using a luminescent assay (CellTiter-Glo^®^ Luminescent Cell Viability Assay, Promega, Madison, WI, USA), which employs a uniform quantitative approach to assess the quantity of viable cells in a culture, relying on the quantification of ATP presence as an indicator of metabolic activity. In brief, a seeding of 5x10^3^ cells was performed in 96-well plates, and these cells were cultured in 50 μL with varying concentrations of TES, 5α-DHT and 5β-DHT for the duration specified in the respective experiment. Subsequently, 50 μl/well of Cell Titer-Glo reagent (comprising Lysis buffer, Ultra-Glo Recombinant Luciferase, Luciferine, and Mg^2+^) was added and gently mixed for 15 minutes at room temperature. Luminescence was then measured using a Glomax Multidetection System (Promega). The results represent three independent experiments performed in triplicate.

### Reagents

The following compounds were obtained from Sigma (St. Louis, MO, U.S.A.): TES (17β-hydroxy-4-andosten-3-one), 5α-DHT (17β-hydroxy-5α-androstan-3-one), acetylcholine chloride (ACh), L-Norepinephrine hydrochloride (NA), sodium nitroprusside (SNP), KCl and HE. The 5β-DHT (17β- hydroxy-5β-androstan-3-one) was obtained from Steraloids (Newport, RI, U.S.A.). The androgens were prepared separately as a stock solution (0.1 M) in absolute ethanol and then diluted in absolute ethanol to 1 mM and then diluted in KHS to the concentration required for each experiment; final ethanol concentration in the tissue baths never exceeded 0.1% (v/v) of the vehicle. The remaining drugs solutions were prepared in distilled water, except for NA which was dissolved in NaCl (0.9%)-ascorbic acid (0.01% w/v) solution. The appropriate dilutions were performed the days of the experiments diluting all the drugs in KHS solutions except for the HE which was diluted in 4-(2-hydroxyethyl)-1-piperazineethanesulfonic acid (HEPES) buffer containing (in mM): 119 NaCl, 20 HEPES, 1.2 CaCl_2_, 4.6 KCl, 0.4 KH_2_PO_4_, 1 MgSO_4_, 5 NaHCO_3_, 5.5 glucose, and 0.15 Na_2_H_2_PO_4_. In addition, SNP and HE were kept in the dark until use to avoid light-induced degradation.

### Statistical analysis

The software GraphPad Prism (GraphPad Software, RRID: SCR_002798) was used to statistically test the results and to generate the graphs. Results are given as mean ± SEM (Standard Error for the Mean) and, firstly, a Shapiro-Wilk test of normality was performed in every group of results to check the normality of the data. In order to compare the effect of androgens on the contraction-response curves, two-way analysis of variance (ANOVA) was performed. To the comparisons of superoxide anion and nitrite release, Student’s *t*-test for unpaired experiments was performed in the cases of parametric data distributions, and Mann-Whitney U test in non-parametric data. A similar analysis was used for the cell proliferation results. In all the experiments, all *p*-values less than 0.05 were considered significant.

## Results

### Different parameters of the experimental group

The weight of the animals before orchidectomy was of 332.5 ± 10.8 g, reaching a value of 689.4 ± 24 g before euthanization (*p* < 0.001). Similar results were observed for the systolic blood pressure (141.5 ± 1.55 and 154.3 ± 2.65 mmHg; *p* < 0.01). The serum level of testosterone was strongly decreased (216 ± 64 pg/mL) respect to previous studies performed in young rats with intact-gonadal function [3].

### Effect of androgens on vascular function

#### Vasodilator responses

**Fig 1:**
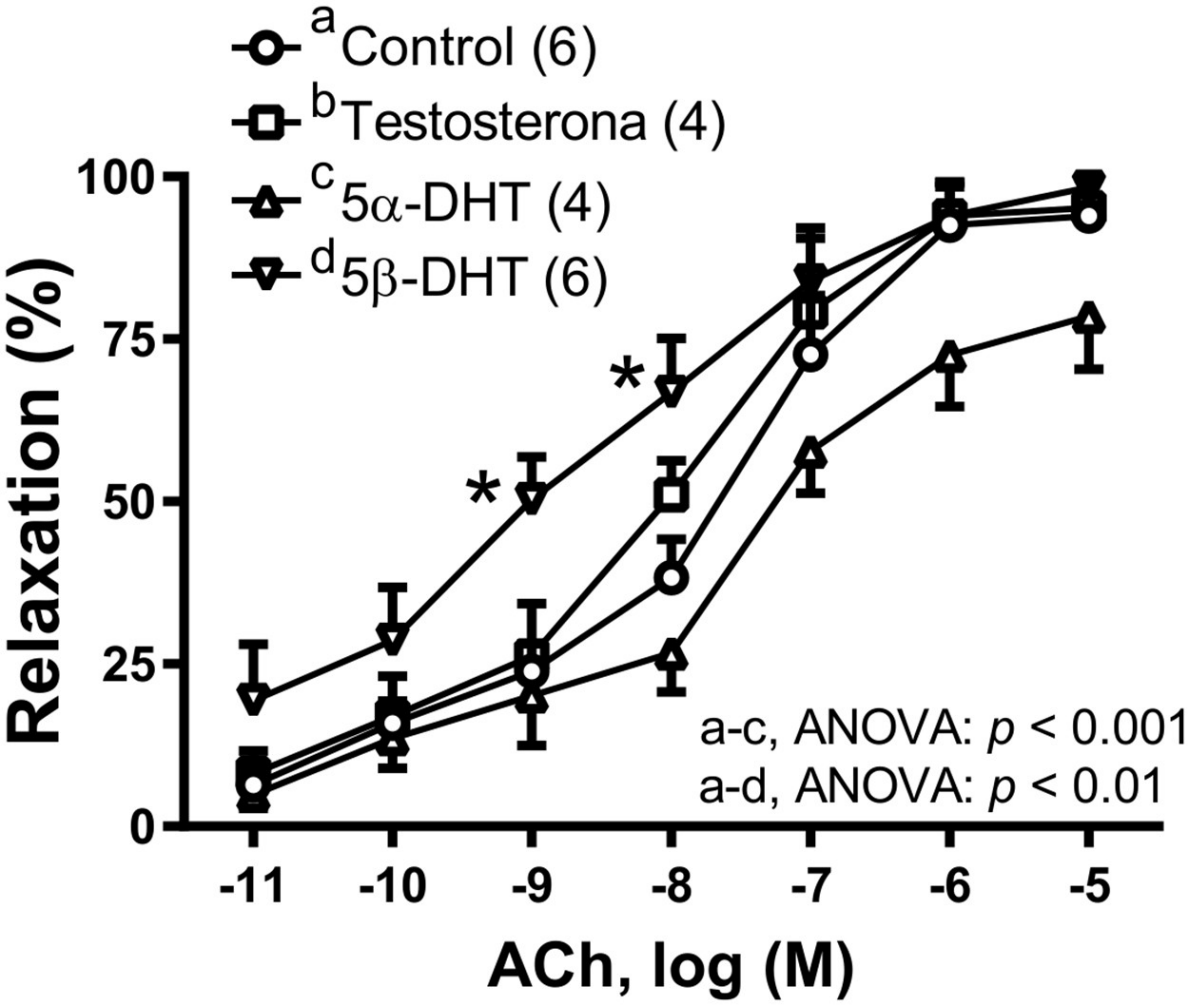
Effect of androgens on the vasodilator response induced by acetylcholine (ACh). Effect of 10 nM testosterone (TES), 5α- and 5β-dihydrotestosterone (5α-DHT and 5β-DHT, respectively) on the concentration-response curves to ACh in the mesenteric arteries of aged-orchidectomized rats. Results (mean ± SEM) are expressed as the percentage of the inhibition of the contraction induced by 1 μM noradrenaline. Number of animals is indicated in parenthesis. The statistical significance is indicated in the graph. * *p* < 0.05 compared with control.

To analyze the effect of androgens on the endothelium-dependent vasodilator response, ACh-induced responses were a studied in the presence of each androgen. In NA-precontracted arteries from aged-orchidectomized rats, 10 nM 5β-DHT increased the relaxation induced by ACh, while 5α-DHT decreased, and TES did not modify the ACh-induced response (Fig 1).

**Fig 2:**
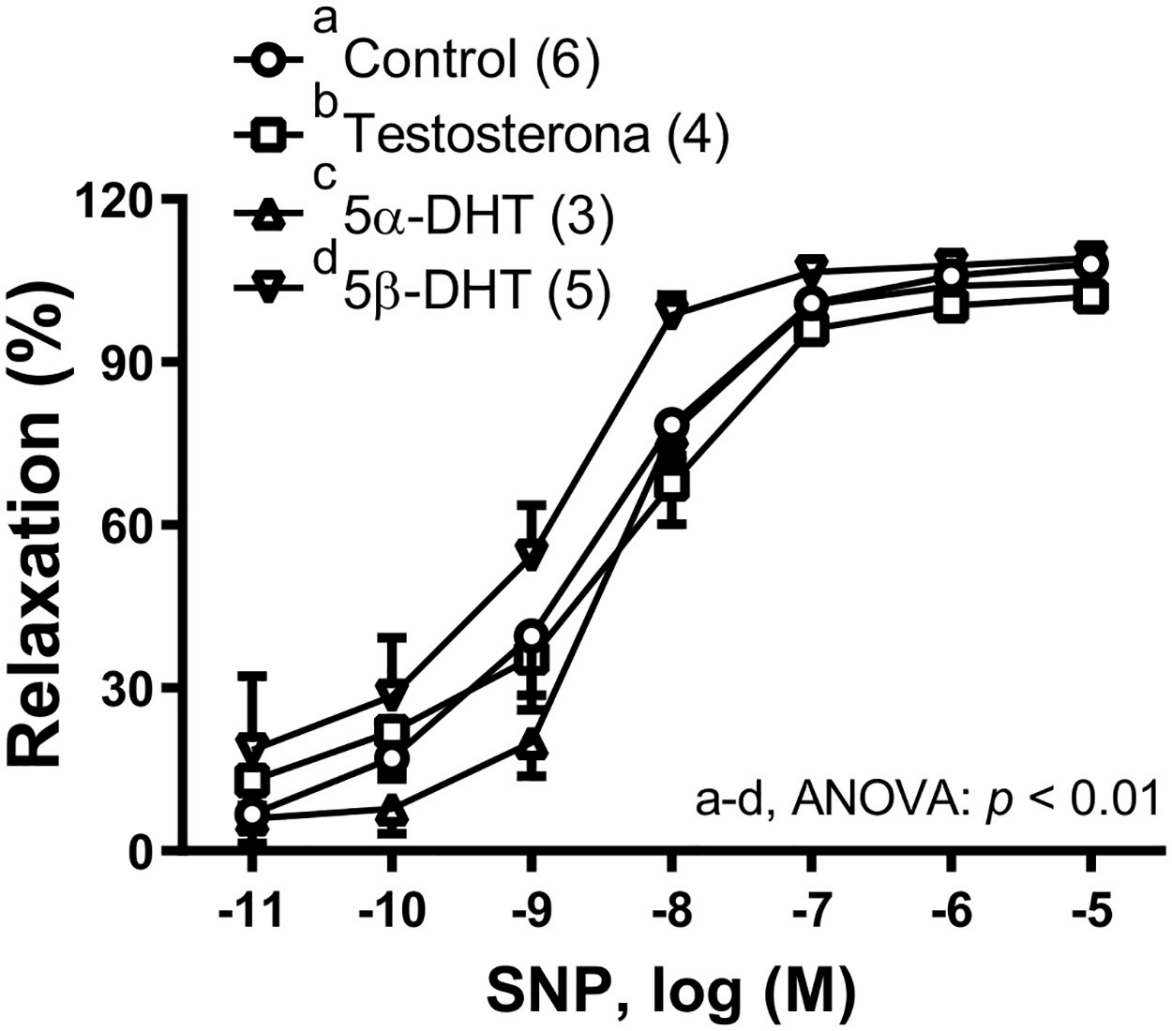
Effect of androgens on the vasodilator response induced by sodium nitroprusside (SNP). Effect of 10 nM testosterone (TES), 5α- and 5β-dihydrotestosterone (5α-DHT and 5β-DHT, respectively) on the concentration-response curves to SNP in the mesenteric arteries of aged-orchidectomized rats. Results (mean ± SEM) are expressed as the percentage of the inhibition of the contraction induced by 1 μM noradrenaline. Number of animals is indicated in parenthesis. The statistical significance is indicated in the graph.

To analyze the possible effect of androgens on the sensitivity of vascular smooth muscle to NO, the vasodilator response induced by the NO donor, sodium nitroprusside (SNP), was studied. In NA-precontracted vessels from aged-orchidectomized rats. Incubation with 5β-DHT increased the SNP-induced relaxation, while incubation with TES or 5α-DHT did not modify the response to SNP (Fig 2).

Although the incubation with the androgens did not modify the basal tone, the NA-induced contraction before performing ACh and SNP curves was analyzed in the four experimental conditions. The results showed that there were no statistical differences in the NA precontraction among groups, therefore the differences observed in the vasodilator responses were due to the actions of ACh or SNP and not to differences in the arterial contraction. For the ACh-curve, the NA-precontraction values were: control = 1160 ± 156 mg; TES = 1113 ± 168.8 mg; 5α-DHT = 1043 ± 57.3 mg; 5β-DHT = 1142 ± 44.3 mg; *p* > 0.05. For the SNP-curve: control = 1515 ± 231 mg; TES = 1521 ± 192 mg; 5α-DHT = 1274 ± 33.2 mg; 5β-DHT = 1565 ± 178 mg; *p* > 0.05. Arteries were exposed to 75 mM of KCl to check their functional integrity (before incubation with the androgens) and the statistical analysis showed no differences among groups: Control = 1497 ± 237.5 mg; TES = 1452 ± 208.2 mg; 5α-DHT = 1210 ± 133.8 mg; 5β-DHT = 1519 ± 73.1 mg; *p* > 0.05).

#### Detection of nitric oxide and superoxide anion

**Fig 3:**
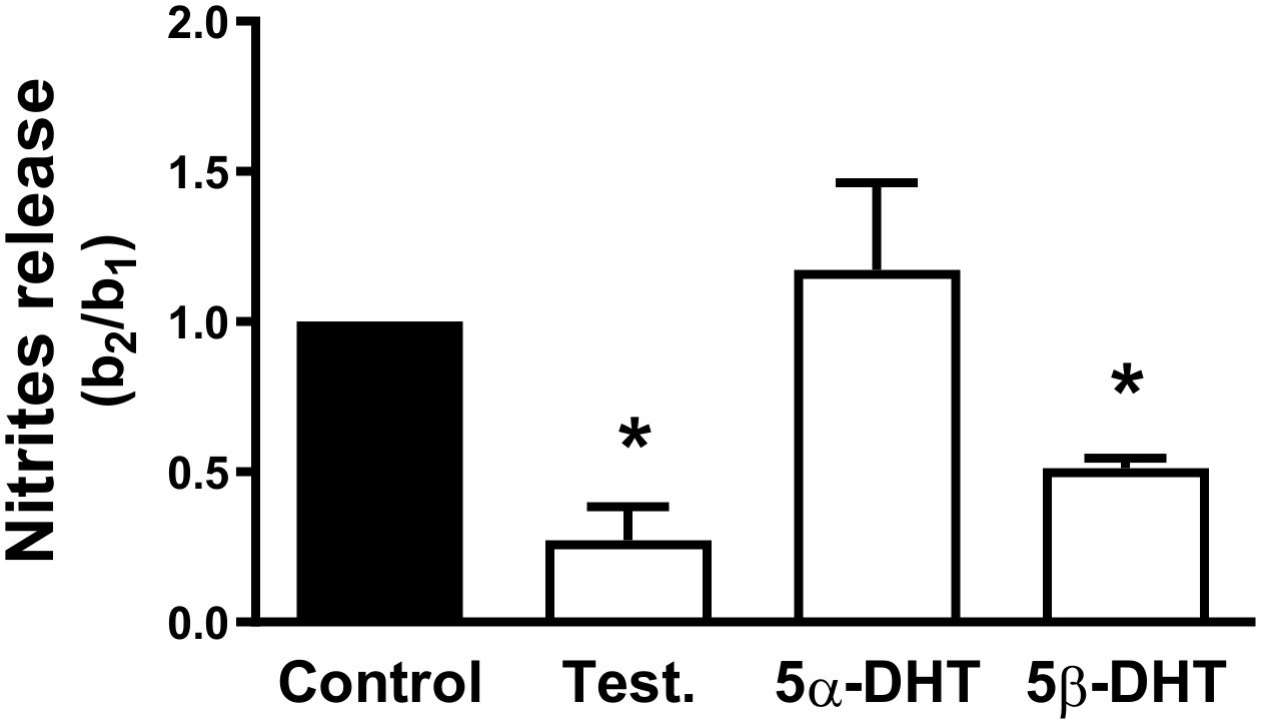
Effect of androgens on the basal NO release. Effect of 10 nM testosterone (TES), 5α- and 5β-dihydrotestosterone (5α-DHT and 5β-DHT, respectively) on the basal release of NO in the mesenteric arteries of aged-orchidectomized rats. Results (mean ± SEM) are expressed as the ratio of the release after (b2) and previous (b1) to the androgens incubation and relative to the control condition (= 1). Number of animals: 4. *p < 0.05 compared with control condition.

The basal release of nitrites was decreased after incubation with 5β-DHT and TES, whereas 5α-DHT did not modify this release (Fig 3).

**Fig 4:**
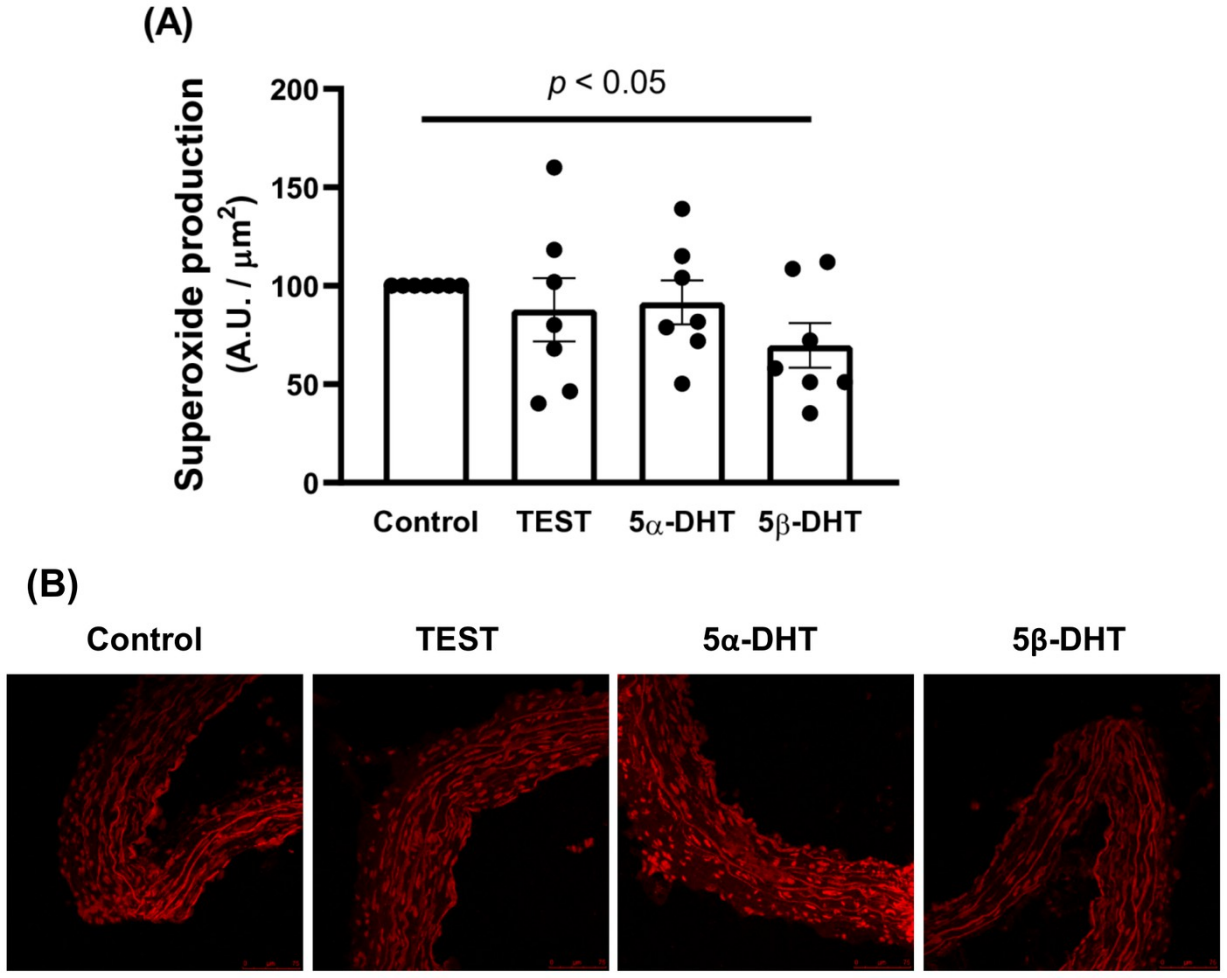
Effect of androgens on the production of superoxide anion. Effect of 10 nM testosterone (TES), 5α- and 5β-dihydrotestosterone (5α-DHT and 5β-DHT, respectively) for 30 min on the production of superoxide anion in the mesenteric arteries of aged-orchidectomized rats (A). Results (mean ± SEM) are expressed as the fluorescence emitted by HE (A.U, arbitrary units) per area (μm2) relative to the control condition (= 100). Number of animals: 3. *p < 0.05 compared with the control condition. Representative images of confocal micrographs (B) showing *in situ* detection of superoxide anion. The statistical significance between 5β-DHT and control condition is indicated in the graph.

Since these results were opposite to those described in previous studies, and it is known that nitrites coming from ROS other than NO can act as substrate for the Griess reaction, the possible effect of androgens on oxidative stress was also analyzed by confocal microscopy. The fluorescence emitted by the fluorescent dye HE in arteries incubated with TES or 5α-DHT was not statistically different respect to the fluorescence in arteries of control condition. Incubation with 5β-DHT decreased the fluorescence emitted by HE in comparison to arteries in control condition (Fig 4).

### Effect of androgens on cell proliferation

**Fig 5:**
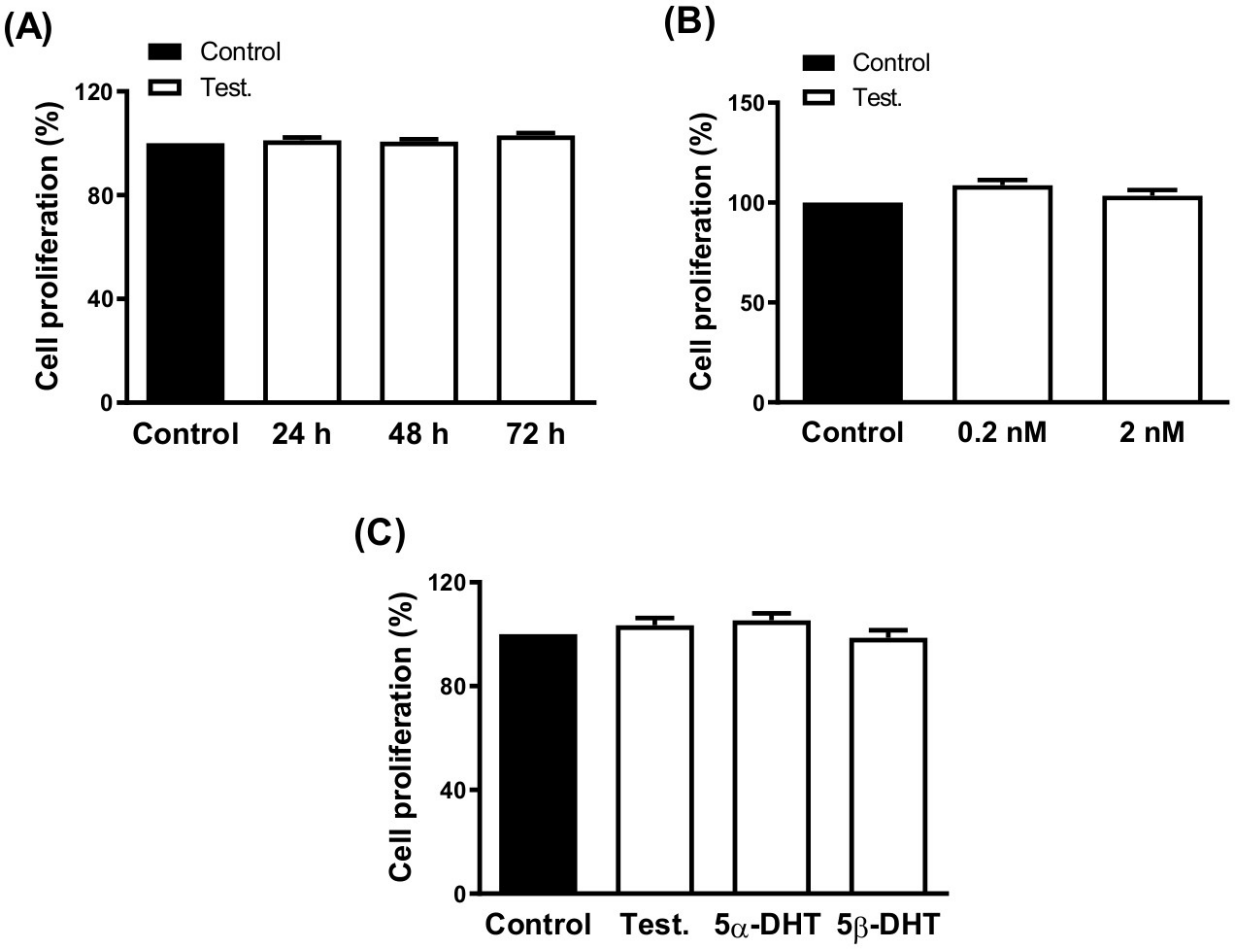
Effect of androgens on the proliferation rate of vascular smooth muscle cells. Effect of incubation with 10 nM testosterone (Test.) for 24, 48 or 72 h (A), 0.2 and 2 nM Test.for 72 h (B) and 2 nM Test., 5α- and 5β-dihydrotestosterone (5α-DHT and 5β-DHT, respectively) for 72 h (C) on the proliferation rate of vascular smooth muscle cells (SV40LT-VSMC). Results (mean ± SEM) are expressed as viability relative to the control condition (= 100). Data represent three independent experiments performed per triplicate.

In a previous study, our research group described that the treatment of cultured VSMC with 10nM TES, downregulated mitogenic signaling pathways induced by the ligand-dependent activation of the epidermal growth factor receptor (EGFR) [5]. Therefore, the effect of 10 nM TES on VSMC proliferation was studied after 24, 48 and 72 h incubation. The results showed that the cell proliferation was not modified after the incubation times analyzed (Fig 5A). Since lower TES concentration has been described to increase smooth muscle cell proliferation [32], the effect of 0.2 and 2 nM TES after 72h exposure was tested. The results showed that under these conditions VSMC proliferation was not modified either (Fig 5B). The use of a hormone-free culture medium did not modify the results above described (data not shown); then, the following experiments were carried out in hormone-free medium. Treatment with 2 nM TES, 5α-DHT or 5β-DHT for 72 h, did not statistically modify the VSMC proliferation (Fig 5C).

**Fig 6:**
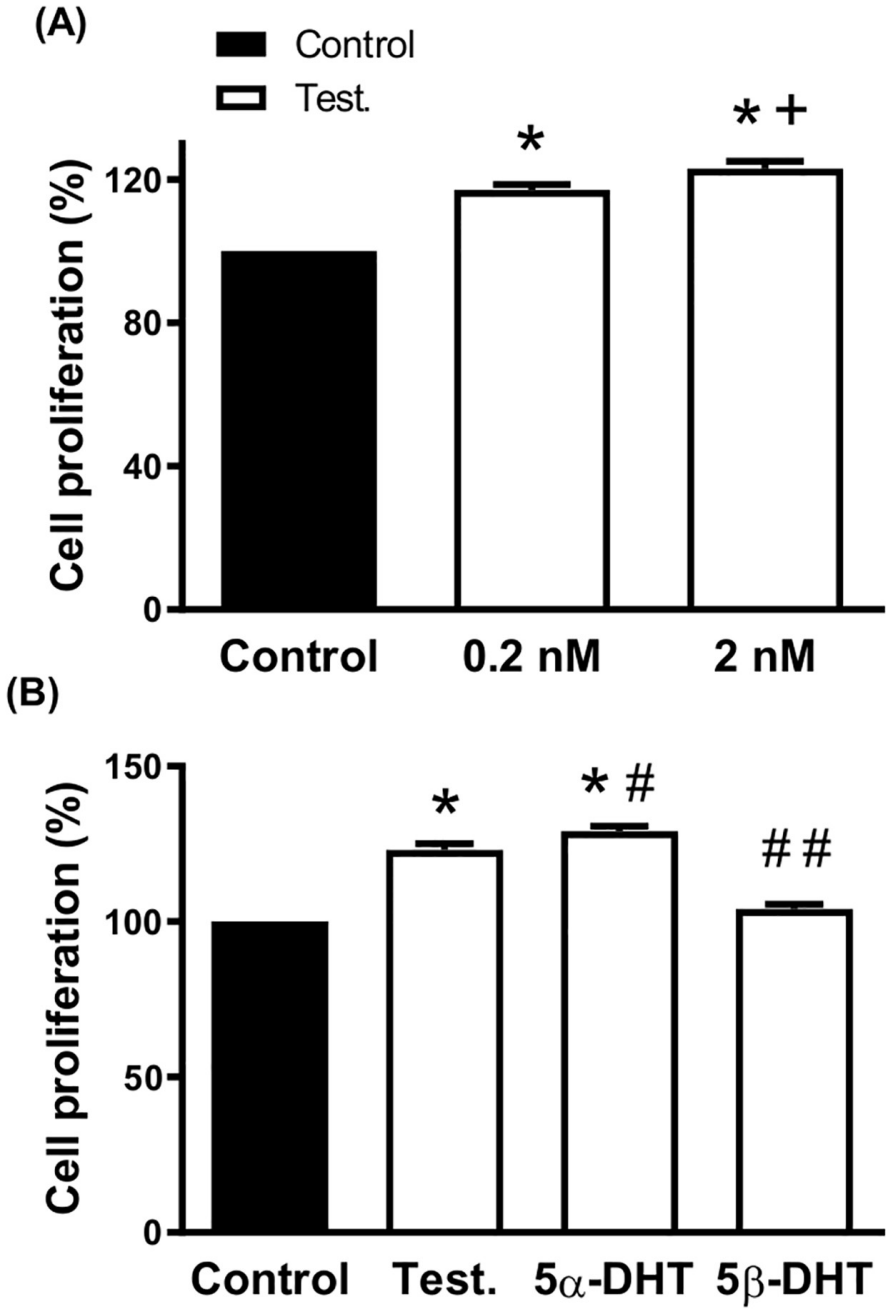
Effect of androgens on the proliferation rate of androgen-dependent prostatic tumor cells. Effect of incubation with 0.2 and 2 nM Testosterone (Test.) for 72 h (A) and 2 nM Test, 5α- and 5β-dihydrotestosterone (5α-DHT and 5β-DHT, respectively) for 72 h (B) on the proliferation rate of androgen-dependent prostatic tumor cells, LNCaP. Results (mean ± SEM) are expressed as viability relative to the control condition (= 100). Data represent three independent experiments performed per triplicate. *p < 0.001 compared with control condition; + *p* < 0.01 compared with 0.2 nM Test.; # *p* < 0.05 compared with Test; ## *p* < 0.002 compared with 5α-DHT.

Regarding the androgen-dependent prostate tumor cells (LNCaP), the proliferative effect of TES was analyzed. The results showed that both 0.2 and 2 nM TES significantly increased the LNCaP cell proliferation, although the increase induced by 2 nM was greater than that of 0.2 nM (Fig 6A). Incubation with 2 nM TES and 5α-DHT for 72 h increased the LNCaP cell proliferation, while 5β-DHT did not statistically modify cell proliferation (Fig 6B).

**Fig 7:**
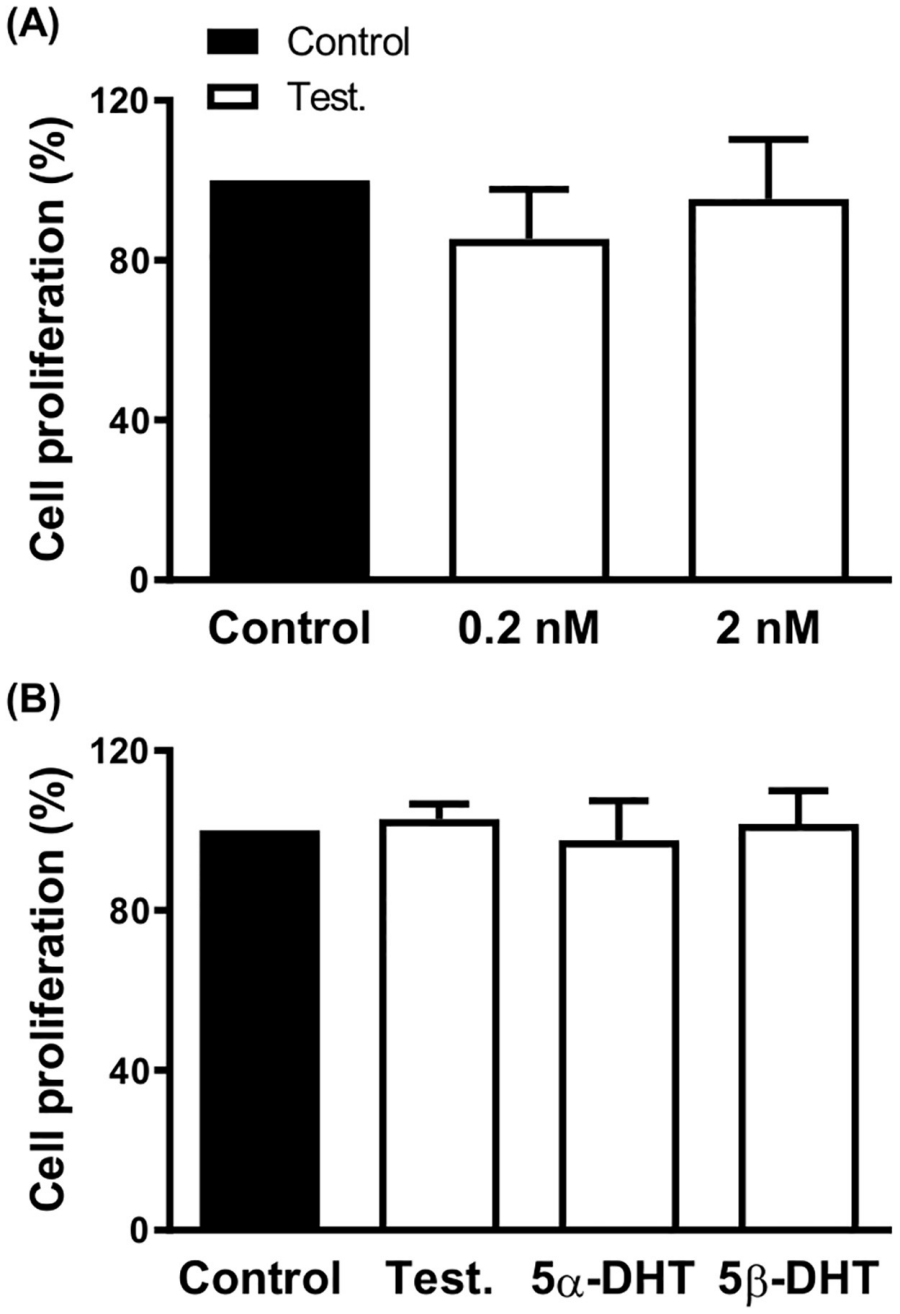
Effect of androgens on the proliferation rate of androgen-independent prostatic tumor cells. Effect of incubation with 0.2 and 2 nM Testosterone (Test.). for 72 h (A) and 2 nM Test., 5α- and 5β-dihydrotestosterone (5α-DHT and 5β-DHT, respectively) for 72 h (B) on the proliferation rate of androgen-independent prostatic tumor cells, DU145. Results (mean ± SEM) are expressed as viability relative to the control condition (= 100). Data represent three independent experiments performed per triplicate.

The effect of 0.2 and 2 nM TES after 72 h exposure was also assessed in the proliferation of the androgen-independent prostatic tumor cells (DU145). The results showed that DU145 cell proliferation was not modified by TES (0.2 and 2 nM) (Fig 7A). After 72 h incubation with 2 nM TES, 5α-DHT or 5β-DHT no modification on DU145 cell proliferation was observed (Fig 7B).

## Discussion

Overall, the current study shows that incubation with 5β-DHT improves vasodilator function of mesenteric arteries from aged-orchidectomized rats which agrees with the *in vitro* beneficial effect of the 5β-reduced metabolite of TES when the vessels present vascular dysfunction. And what is even more important is that 5β-DHT does not increase the proliferation rate of the androgen-dependent prostatic tumor cells, LNCaP. All the results found will be discussed below.

During the physiological process of aging, endothelial dysfunction is associated with a decrease in NO bioavailability and an increase in ROS production, which leads to a gradual decline in functionality of the vascular system [33–36]. Aging is also associated with a decrease in plasma levels of TES [13, 37]. It has been described that androgens exert protective effects on the function of the cardiovascular system since epidemiological studies have shown a correlation between decreased levels of TES and a higher incidence of cardiovascular diseases [6, 7, 38, 39]. On the other hand, the use of testosterone hormone re-placement in patients diagnosed with hypotestosteronemia decreased cardiovascular risk factors [13, 14, 40]. Likewise, studies carried out in animal experimental models have described that the loss of gonadal function alters the release and function of different cells mediators such as NO, prostanoids and ROS. Thus, we have found a decreased NO release in arteries from orchidectomized rats in a time-dependent manner [5] as well as an increase in thromboxane A_2_ (TXA_2_) [3, 4, 41] and ROS production [1, 2, 42]. On the other hand, numerous studies have demonstrated the vasodilator actions of TES and its 5-dihydroreduced metabolites, 5α-DHT and 5β-DHT, [10, 17, 20] that could underly the hypotensive effects observed *in vivo* in different rat strains [23, 43].

The experimental model used in the current investigation, the aged-orchidectomized rat, showed a drastic decrease in the levels of TES which agrees with studies previously reported about orchidectomy [3, 42, 44] or aging [45]. Different studies have demonstrated that both loss of TES and aging process induce alterations in the lipid profile that promotes greater adiposity [42, 44, 46, 47] which would be in line with the body weight observed in the experimental animal group. In addition, the value of systolic blood pressure indicate that is higher than in young animals with the intact gonadal function used in previous studies [3, 42, 44], probably as consequence of the increased oxidative stress described in aging [34, 48] and in orchidectomy [1, 2, 42].

In the current study, the effect of TES, 5α-DHT and 5β-DHT on the endothelium-dependent vasodilation was firstly analyzed. The results showed that 5β-DHT increased the ACh-induced response, while 5α-DHT decreased, and TES did not modify that response in vessels from aged-orchidectomized rats. These results agree with previous studies demonstrating that the greatest effects (both vasodilator and hypotensive effects) were obtained with 5β-DHT -compared with TES or 5α-DHT- in vessels from animal models with cardiovascular pathologies [20, 21, 23].

Regarding the different effect of 5α-DHT and 5β-DHT on the ACh-induced responses in mesenteric arteries from aged-orchidectomized rats the results could be explained by differences in their structural conformation [17, 23]. The decrease in the ACh-induced response by 5α-DHT has been already reported in a rat model of polycystic ovary syndrome [49]. Likewise, the treatment with 5α-DHT in female ovariectomized mice induced arterial stiffness [50] which could be involved in the decreased vasodilator responses. However, to our knowledge, there are no studies comparatively analyzing the effect of 5α-DHT and 5β-DHT on the endothelium-dependent response induced by ACh. The increase in ACh-induced response by 5β-DHT could be related to modifications in the release of different relaxing and contracting factors through muscarinic receptor activation [51]. Since NO is one of the most important relaxing factors released in response to ACh, the smooth muscle sensitivity to NO was analyzed. The vasodilator response induced by the NO donor SNP was increased by 5β-DHT. Similar results were observed in the aorta [52] and mesenteric artery [21] from hypertensive rats.

The results showed that TES and 5β-DHT decreased the basal nitrite release while 5α-DHT did not significantly modify that release. Surprisingly, these results are apparently opposite to those previously described, which showed that 5β-DHT increased the neuronal NO release in arteries from normotensive rats [21] and that the loss of gonadal function for 6 weeks decreased the endothelial NO release [5]. In addition to these results, it is important to keep in mind what has previously been described: (i) a decreased of the NO bioavailability in cardiovascular pathologies associated to aging [34, 48]; and (ii) the oxidation of tetrabiopterin, an eNOS cofactor, could occur in arteries with an increased oxidative stress leading to an eNOS uncoupling [53, 54]. Uncoupled eNOS can synthesize a substantial amount of superoxide anion and, in turn, peroxynitrite [55], which is metabolize to nitrite and is substrate for the Griess reaction [56, 57]. Therefore, the determined nitrite levels could come from the metabolism of NO as well as from other nitrogen species, such as peroxynitrite. Based on this information and that the antioxidant effect of TES has been described [58, 59], the effect of the androgens on the superoxide anion production was investigated. The confocal microscopy experiments showed that TES and 5α-DHT did not significantly modify the superoxide production. Incubation with 5β-DHT reduced superoxide anion production compared to the control condition. These results show that 5β-DHT has an antioxidant effect, which matches with that found in aorta of hypertensive rats [52] that could be involved in the increased vasodilator function, probably through increasing NO bioavailability. Nevertheless, the effect of androgens on the release of factors other than NO cannot be ruled out. Thus, preliminary experiments showed that the release of TXA_2_ tends to be increased after incubation with 5α-DHT, which could contribute to the decreased ACh-induced response observed in our experimental conditions. Thus, increased TXA_2_ release involved in the decreased ACh-induced response has been described in arteries from rats treated with 5α-DHT for three months [49]. Likewise, it would be interesting to investigate the possible modification in the contribution to hyperpolarizing mechanisms by the androgens, since they also act on potassium channels. Although the participation of TXA_2_ and hyperpolarizing mechanisms was not analyzed, which may represent a limitation of the study, our results open a series of future studies on the mechanisms underlying the differential effects of androgens. Additionally, *in silico* experiments would be an interesting approach to elucidate how androgens interact with proteins to exert their actions.

Apart from these mechanistic studies that will be carried out in future research, the results obtained on the vasodilator function continue to support the potential use of 5β-DHT as therapeutical agent for the treatment of different cardiovascular pathologies. Although the non-genomic action of 5β-DHT has been described [60], it would be essential to verify the possible effect of 5β-DHT on the proliferation rate of prostatic tumor cells since an important number of actions initiated on membrane proteins can ultimately modulate the expression of different genes and modify cell proliferation. Also, the analysis of the effect of androgens on the proliferation rate of VSMC would be of relevance since this action could lead to vascular remodeling. Moreover, considering that testosterone deficiency increased vascular remodeling [50, 61]. In a previous study 10 nM TES decreased the activation of the EGFR in VSMC and in mesenteric arteries from orchidectomized rats [5]. Based on this information, the effect of 10 nM TES on the proliferation rate of VSMC was tested. The results showed that incubation with TES for 24, 48 or 72 h did not modify the cell proliferation rate. It is well known that oxidative stress is involved in the cell proliferation process [62]. However, we have above described that 5β-DHT decreased the superoxide formation but did not modify the VSMC proliferation. It is important to mention that the results observed as consequence of androgens incubation may depend on the experimental system models. Thus, the antioxidant ability of 5β-DHT was showed in arteries from aged-orchidectomized rats, in which an overproduction of superoxide already occurs. On the other hand, the action of androgens in VSMC proliferation was analyzed in an established cell line without simulating aging process. Taking into account that both proliferative [63] and antiproliferative [64, 65] effects of TES on VSMC have been described, the effect of lower concentrations of TES, i.e. 0.2 and 2 nM, for 72 h was analyzed. The results pointed out that TES, at 0.2 and 2 nM, also did not modify the proliferation of VSMC. These discrepancies could be due to differences in cell lines and/or in experimental protocols used. Considering these results, the effect of 0.2 and 2 nM TES was analyzed on prostatic tumor cells, both in androgen-dependent and -independent. The results showed that 0.2 and 2 nM TES increased the proliferation rate of LNCaP cells while did not modify the proliferation rate in the androgen-independent DU145 cells, which agrees with that previously described [66–68].

An important number of investigations have described the effect of TES and 5α-DHT on the growth of tumor prostatic cells. However, comparative studies analyzing the effect of 5β-DHT are lacking. The results described in the current study pointed out that none of the androgens (2 nM, for 72 h) modified the proliferation rate of VSMC, which apparently disagreed with the inhibitory effect of TES on vascular remodeling of rat mesenteric arteries [4, 5]. Nevertheless, it is important to keep in mind the notable differences among the experimental models. With respect to the effect of androgens on prostatic tumor cells, neither of the androgens had any effect on the proliferation rate of DU145 cells, as would be expected since the growth of these cells is considered androgen independent [68]. On the other hand, the proliferation rate of LNCaP cells was increased by the incubation with 2 nM TES and 5α-DHT for 72 h, in agreement with previous results [67, 69]. What is of special relevance is that 5β-DHT -in contrast to 5α-DHT- did not induce any modification on the proliferation rate of LNCaP cells.

Taking together all the results, 5β-DHT could be considered a potential drug for the treatment of cardiovascular diseases associated with pharmacological loss of androgens -in prostate cancer patients under ADT [9]- as well as non-pharmacological induction as occur in diabetes [70]. In this sense, 5β-DHT has been administered for 5 weeks to hypertensive rats -whose hypertension had been induced by orchidectomy- demonstrating its antihypertensive effect [71]. However, extensive basic research still needs to be performed to demonstrate the *in vitro* and *in vivo* long-term effects of 5β-DHT on vascular function and on prostate tumor progression. Thus, information obtained with *in vivo* models will be particularly important, since the model mimics the complex cellular interactions that occurs in the tumor microenvironment in the whole organism.

In conclusion, and although a deeper knowledge of the mechanisms of action of 5β-DHT are necessary, two findings from this study deserve to be highlighted: (i) the fact that 5β-DHT does not increase the proliferation rate of prostatic tumor cells, and (ii) its regulatory capacity for improving vascular function. All this makes 5β-DHT a promising therapeutic agent for the treatment of cardiovascular pathologies.
